# Psychological and neurophysiological measures of emotion dysregulation in borderline personality disorder and posttraumatic stress disorder

**DOI:** 10.1186/s40479-025-00313-3

**Published:** 2025-09-10

**Authors:** Isabelle Göhre, Sarah Back, Simone Schütz-Bosbach, Qiaoyue Ren, Larissa Wolkenstein, André Rupp, Katja Bertsch

**Affiliations:** 1 Department of Psychology, Ludwig-Maximilians-Universität München, Leopoldstr. 13, Munich, 80802 Germany; 2 German Center for Mental Health (DZPG), partner site Munich, Munich, Germany; 3 University Hospital Heidelberg, Heidelberg, Germany; 4 Department of Psychology, Julius-Maximilians-Universität Würzburg, Würzburg, Germany

**Keywords:** Trauma-related disorders, Emotion regulation, Emotional reactivity, Childhood trauma, Event-related potentials

## Abstract

**Background:**

Emotion dysregulation is a central feature in trauma-associated disorders such as posttraumatic stress disorder (PTSD) and borderline personality disorder (BPD). However, it remains unclear whether emotion dysregulation is a transdiagnostic phenomenon closely linked to childhood trauma, or if disorder-specific alterations in emotion processing exist. Following a multimethodological approach, we aimed to assess and compare the reactivity to and regulation of emotions between patients with BPD and PTSD, as well as healthy controls, and identify associations with childhood trauma.

**Methods:**

A total of 135 women, 43 healthy controls, 43 with BPD and 49 with PTSD, took part in a multimethodological assessment of emotional reactivity and regulation. Self-report measures were used to assess childhood trauma and emotion dysregulation. Additionally, participants performed a classic emotion regulation (ER) paradigm. Subjective emotional valence ratings and neurophysiological responses (P3 and late positive potential, LPP) were measured in response to negative, positive, and neutral pictures (emotional reactivity) and during active regulation vs. passive viewing of negative pictures (ER).

**Results:**

Regarding emotional reactivity, during the experimental paradigm both patient groups reported lower emotional valence after viewing positive or neutral pictures compared to healthy controls. Furthermore, P3 amplitudes in response to neutral pictures were reduced in both patient groups and in response to negative pictures, specifically in patients with PTSD. Regarding ER, while both patient groups self-reported significant disturbances in ER, neither valence ratings nor neurophysiological responses assessed during the ER task (P3, LPP) differed from healthy controls. Across groups, childhood trauma was related to decreased emotional valence ratings on neutral and positive pictures and higher self-reported emotion dysregulation.

**Conclusions:**

Patients with BPD and PTSD exhibited a reduced emotional reactivity in response to positive and neutral information. Specifically, patients with PTSD demonstrated hypo-reactivity to neutral and trauma-unrelated negative stimuli, which might be due to altered attentional resource allocation following trauma. Although patients reported using adaptive ER strategies less frequently in daily life, they effectively implemented them when instructed to, highlighting important clinical and theoretical implications.

**Supplementary Information:**

The online version contains supplementary material available at 10.1186/s40479-025-00313-3.

## Introduction

Emotion dysregulation is a key feature of various trauma-associated disorders, including borderline personality disorder (BPD) and post-traumatic stress disorder (PTSD) [1, 2]. Especially traumatic experiences in childhood can result in psychosocial and neurobiological alterations in emotion processing [3–5]. Two key components influenced at different stages of emotional processing are: *emotional reactivity* and *emotion regulation (ER)* [6]. Emotional reactivity refers to the implicit response to emotionally salient stimuli and encompasses changes in subjective experience, behaviour and physiology [2]. According to Gross’s process model, ER describes the complex processes by which individuals influence which emotions they experience, when they experience them, and how they express and respond to them [7, 8]. Therefore, ER strategies can be applied at different stages of the emotion-generative process, either before (antecedent-focused, e.g., cognitive reappraisal) or after (response-focused, e.g., emotion supression) an emotional reactivity has occurred. Clinical frameworks, such as Linehan’s biosocial model of BPD [5], further support that deficits can occur at different stages, from heightened emotional sensitivity/reactivity to deficits in the use of adaptive regulation strategies such as antecedent-focused, cognitive reappraisal [2].

While both BPD and PTSD are marked by alterations in emotion processing, their primary diagnostic features suggest that they differ in their emotional reactivity and regulation. Individuals with BPD often display pronounced emotional *hyper-reactivity* (i.e., emotional instability) alongside an *under-regulation* of emotional reactions with deficit in adaptive strategies and a surplus of maladaptive strategies such as self-harm [2, 9]. Individuals with PTSD may also exhibit an emotional *hyper-reactivity* (i.e., hyperarousal) to emotional threats but often attempt to *over-regulate* distress stemming from traumatic memories through emotional avoidance or numbing [10]. Despite these distinctions, direct comparisons of emotional reactivity and regulation in BPD and PTSD remain scarce [11, 12]. This represents a critical gap, since both disorders share significant overlap in their etiological underpinnings - such as traumatic experiences in childhood - and in their symptomatic manifestations of emotion dysregulation [3–5]. Therefore, it remains unclear whether emotion dysregulation reflects a transdiagnostic phenomenon closely linked to childhood trauma, or if disorder-specific alterations in emotion processing exist. Clarifying this distinction is crucial for improving the clinical differentiation and informing the development of transdiagnostic models and interventions targeting emotion dysregulation.

Another limitation of existing research is its reliance on single-method approaches, such as self-reports or experimental tasks, which fails to capture the multidimensional nature of emotional reactivity and regulation across psychological, behavioral, and neurophysiological domains. A multimethodological approach - integrating self-reports, experimental paradigms, and neurophysiological data - provides a more comprehensive understanding of these processes. Revealing discrepancies between subjective and objective measures can guide accurate assessments and improve clinical interventions that aim to target a specific ER process.

In summary, there is a pressing need for research that systematically compares emotional reactivity and regulation among individuals with BPD and PTSD using a multimethodological approach. In the following sections, we will first outline different methods for assessing emotional reactivity and regulation, including self-reports, experimental paradigms, and neurophysiological data. Next, we will provide an overview of the current knowledge on emotion processing in BPD and PTSD patients based on these methods. We will then explore knowledge on the transdiagnostic link between childhood trauma and emotion processing in BPD and PTSD and conclude by outlining our research aims based on these insights.

### Methods for assessing emotion processing

Self-report questionnaires, like the Difficulties in Emotion Regulation Scale (DERS) [13], assess dysfunctions in emotion processing, including the awareness, clarity, and acceptance of emotional reactions as well as the use of adaptive ER strategies. As proposed by McRae [14], questionnaires aim to measure the *frequency* of using ER strategies flexibly in everyday life over the *long-term*. In contrast, experiments measure the *short-term* emotional reactivity to stimuli (e.g., positive, negative and neutral pictures) and the *success* of employing an instructed ER strategy in the laboratory. For example, one experimentally measurable ER strategy is *cognitive reappraisal*, which e.g., involves reinterpreting the meaning of a negative picture to reduce its emotional impact [15]. The emotional reactivity to pictures and the effect of the ER strategy can be measured on multiple levels, including subjective ratings of emotional valence and objective neurophysiological responses. Other modalities, such as behavioral or physiological measures (e.g., heart rate, pupil dilation), can also provide valuable insights.

The neural activity in response to emotional pictures can be assessed through event-related brain potentials (ERPs) [16]. Among other ERP components, the P3 and the Late Positive Potential (LPP) are particularly recognized as reflecting cortical emotion processing [17]. The centroparietal P3, a positive deflection around 300 to 500ms following picture presentation, exhibits enhanced amplitude in healthy individuals in response to emotionally salient (i.e., negative and positive) compared to neutral pictures [16]. The P3 is thought to represent conscious attentional processing of emotional information and resembles the early phase of the LPP. The LPP, a positive wave extending up to 1500ms post-stimulus, is associated with more conscious processing of emotionally significant pictures with intrinsic motivational relevance [18]. As ER processes typically require more time to develop, they might primarily impact later components, such as the LPP [19]. In healthy individuals, cognitive reappraisal of negative pictures was associated with decreased LPP amplitudes compared to passive viewing [19–22].

### Emotion processing in BPD and PTS

#### Emotional reactivity

Self-report studies indicate heightened negative emotional reactivity in individuals with BPD [23–25] and PTSD [26–28]. However, experimental findings on valence ratings in response to emotional pictures remain inconsistent. Studies have found that individuals with BPD [29–31] and PTSD [32] report lower positive valence (hypo-reactivity) after exposure to (positive and) neutral pictures compared to healthy volunteers. However, findings on valence ratings after negative pictures have yielded mixed results. Studies using *trauma-unrelated negative pictures* often find no discernible differences from healthy controls in PTSD patients [33, 34] and BPD patients [29]. However, studies using *trauma-related negative pictures* report heightened negative valence ratings in individuals with PTSD [32, 35, 36] and BPD [37]. This might suggest that negative emotional hyperreactivity in BPD and PTSD is not generalized but context-dependent, particularly for pictures depicting trauma-related themes, such as childhood abuse, abandonment or social rejection [29, 38–40].

Research on P3 and LPP amplitudes in response to emotional pictures in BPD and PTSD is scarce, with existing findings remaining highly inconsistent and difficult to compare due to methodological and analytical differences [41–43]. One study has found elevated LPP amplitudes in response to negative compared to neutral pictures in patients with BPD [30]. However, studies that compared the amplitude to negative, positive or neutral pictures separately, found no difference compared to healthy controls [29, 44]. Moreover, studies using pictures of emotional faces have found reduced P3 and LPP responses compared to HC [45], particulalry for positive (i.e., happy) faces [46, 47]. A similarly inconsistent pattern emerges for PTSD, with some studies indicating a reduced P3 amplitude in response to trauma-unrelated negative or neutral pictures compared to HC [34, 36, 41], while others find an increased P3 amplitude to trauma-related pictures [35]. In contrast, several studies report no significant differences to HCs in the P3 amplitude [33, 48] or LPP amplitude [33, 48–50].

#### Emotion regulation

Reviews and meta-analyses of self-report studies suggest that individuals with BPD exhibit deficiencies in the *frequency* of attenuating intense emotions in every-day life through adaptive ER strategies such as cognitive reappraisal [24, 51]. Individuals with PTSD often self-report general emotion dysregulation, characterized by difficulties in reducing emotional reactivity and less frequent use of adaptive ER strategies [1, 52].

In contrast, findings from experimental studies have not reliably demonstrated difficulties in ER in both BPD [30, 44, 53] and PTSD [50]. Several studies report no significant difference between patients and healthy controls in ER tasks. When instructed to, individuals with BPD [29, 51, 54] and PTSD [50] might be able to employ cognitive reappraisal to down-regulate negative valence ratings. Moreover studies in BPD [30] and PTSD patients [49, 50] have found no difference to HCs in the effect of cognitive reappraisal on LPP amplitudes.

### Childhood trauma and emotion dysregulation

Explanatory models of emotion dysregulation in trauma-associated disorders, such as BPD and PTSD, propose childhood trauma as a shared etiological mechanism [3–5]. Childhood trauma - encompassing sexual, physical, and emotional abuse as well as physical and emotional neglect before the age of 18 years - can impact emotion processing on both psychosocial and neurophysiological levels in BPD and PTSD [3, 31, 55].

On a psychosocial level, abuse and neglect can result in insecure or disorganized attachments due to a lack of secure emotional bonding [56, 57]. Due to the caregivers invalidating and abusive reactions to emotional needs, a child might cope with a hyperreactivity to threat-related socio-emotional cues [58] and maladaptive ER strategies (e.g., avoidance) [59]. On a neurobiological level, childhood trauma might be associated with increased threat sensitivity, driven by limbic hyperactivation (e.g., amygdala, hypothalamus-pituitary-adrenal axis) alongside impaired regulation by fronto-cortical circuits [55, 60, 61].

Despite these well-documented effects of childhood trauma on emotion processing, its influence on experimental measures of emotional reactivity and regulatios, such as valence ratings and neurophysiological responses, remains underexplored in BPD and PTSD. In BPD, more severe childhood trauma experiences and lower parental attachment has been linked to greater emotional reactivity to positive pictures in valence ratings [31]. Moreover, a study in BPD patients found that childhood trauma was correlated with ERP amplitudes in response to pictures depicting neutral, physical painful, and psychological painful interactions during a “Social Interaction Empathy Task” [62]. In contrast, studies in healthy adults [63] and university students [64] have reported a link between childhood trauma and reduced ERP amplitudes in response to unpleasant pictures, with stronger effects in individuals with lower top-down impulse control. Similarly, in trauma-exposed adolescents, a higher number of traumatic events correlated with a reduced ERP difference between positive and neutral IAPS pictures [65]. The authors interpreted this blunted response as reduced affective discrimination or desensitization to intense emotions after childhood trauma. Importantly, an intervention study that targeted emotional coping strategies in adolescents with childhood trauma highlighted the potential to improve affective discrimination in form of increased LPP responses [66].

These findings underscore the need to examine the transdiagnostic role of childhood trauma in psychosocial and neurophysiological emotion dysregulation in BPD and PTSD to inform the development of clinical interventions. In this regard, it is crucial to determine whether childhood trauma serves as one of the primary transdiagnostic factors driving emotion dysregulation in BPD and PTSD that provides explanatory value beyond traditional diagnostic boundaries.

### The present study

Although emotion dysregulation is a hallmark feature of BPD and PTSD, it remains unclear whether it represents a transdiagnostic consequence of childhood trauma or involves disorder-specific alterations, such as differences in the intensity of emotional reactivity or the capacity for emotion regulation unique to each disorder. Existing research lacks direct comparisons of the disorders and a multimethod approach. To address this, we assessed emotion processing using self-reports as well as emotional valence ratings and neurophysiological responses (ERPs) in an experimental task in female patients with BPD, female patients with PTSD, and healthy women. Emotional reactivity was measured through responses to positive, neutral, and negative images, while ER was assessed by comparing affective responses during cognitive reappraisal versus passive viewing of negative images.

Based on existing literature, we hypothesized (1) elevated emotional reactivity in the experimental task, operationalized as lower valence ratings and increased P3 and LPP amplitudes in response to negative emotional stimuli in individuals with BPD and PTSD compared to healthy controls. (2) The instruction to down-regulate negative emotions (reappraisal vs. view condition) was hypothesized to result in a smaller increase in valence ratings and a lower decrease in late LPP amplitudes in individuals with BPD and PTSD compared to healthy controls, indicating less effective downregulation. (3) We expected significantly elevated self-reported emotion dysregulation, as measured by the total score and subscales of the DERS, in patients with BPD and PTSD compared to healthy controls. (4) We expected childhood trauma, as measured by the total score of the Childhood Trauma Questionnaire (CTQ), to be associated with alterations in emotional reactivity and regulation across diagnostic categories, suggesting a potential transdiagnostic relevance. (5) Due to a lack of previous studies, no a priori hypotheses could be formulated concerning the differences between BPD and PTSD and therefore exploratory comparisons were conducted. Since emotion dysregulation is the core symptom of BPD [5], one may expect that individuals with BPD would show more pronounced alterations in emotional reactivity and regulation than those with PTSD.

## Methods

### Participants

Forty-three women with BPD, fourty-nine women with PTSD and forty-three healthy women were included in this study. Exclusion criteria comprised age < 18 or > 50 years; a body-mass-index < 17 or > 30; insufficient proficiency in the German language; chronic medical illness, including cardiovascular abnormalities; substance abuse or dependency in the past six months; and a lifetime diagnosis of schizophrenia, schizoaffective or bipolar disorder. HC had never received a psychiatric diagnosis or undergone a psychotherapeutic/psychiatric treatment. Patients with BPD had to have a primary DSM-5 diagnosis of BPD (≥ 5 diagnostic criteria); patients with PTSD had a primary DSM-5 diagnosis of PTSD but did not fulfil ≥ 3 diagnostic criteria of BPD.

Parts of the study were assessed within a larger preregistered project (preregistration at www.drks.de: DRKS00019945), which originally included the recruitment of HC and individuals with BPD and specified analyses focusing on interoception as possible mediator between childhood trauma and ERP-based emotion regulation. The current analyses, which include the recruitment of a PTSD group as well as group comparisons of multiple ER measures (self-report and experimental variables), and the hypothesis regarding transdiagnostic effects of childhood trauma, were however not part of the preregistration. Participants were recruited by referral of practitioners and hospitals, self-help organizations, and advertisements (e.g., in newspapers and social media). The study was performed in accordance with the principles of the Declaration of Helsinki and approved by the ethics committee of the Department of Psychology, LMU Munich. Participants provided written informed consent and were financially reimbursed.

### Experimental protocol

For all participants, the diagnostic process comprised an extensive telephone screening for inclusion and exclusion criteria (approx. 45 min) followed by an onsite diagnostic appointment (approx. 3 h; see below) and the experimental session (approx. 2 h). Before the experiment, participants underwent a urine toxicology screening to exclude acute substance abuse. They filled out several state questionnaires and were prepared for the EEG measurement which took place in a sound-proofed and electrically shielded laboratory. Self-report questionnaires were filled out digitally either at home or in the laboratory prior to the experiment.

### Diagnostic and self-report measures

Onsite diagnostic appointments consisted of three interviews performed by experienced diagnosticians with at least a M.Sc. in psychology and clinical training. The International Personality Disorder Examination - Borderline Section [IPDE-BOR, [67]] is a semi-structured interview that allows both a categorical BPD diagnosis and a dimensional assessment of symptom severity according to DSM-IV criteria. A diagnosis was made if five or more of the nine criteria were met, based on 15 items rated as 0 = “absent/normal”, (1) “exaggerated/accentuated” or (2) criterion level/pathological, yielding a total score range of 0–30. The IPDE-BOR has demonstrated a high interrater reliability compared to other diagnostic tools [68]. The Clinician-Administered PTSD Scale for DSM-5 [CAPS-5, [69]] was used as a structured interview to diagnose PTSD and assess symptom severity. The clinician-assigned scores range from 0 = “absent” to 4 = “extremely”, based on a combination of the frequency and intensity of the 30 DSM-5 symptoms, with a total score range of 0–120. The German version of the CAPS has shown a high internal consistency and inter-rater reliability in a trauma-exposed sample [70]. Lastly, the Structured Clinical Interview for DSM-5 Disorders – Clinicial Version [SCID-CV, [71]] was included as a semi-structured diagnostic tool for the assessment of comorbid Axis-I disorders according to DSM-5. The SCID-CV has demonstrated a high clinical validity and inter-rater reliability [72].

For the assessment of childhood trauma and emotion dysregulation the following self-report questionnaires were used:

The German version of the Childhood Trauma Questionnaire (CTQ) [73] was used to retrospectively assess childhood trauma before the age of 18 years. Its 28 items (a five-point Likert scale ranging from 1 = “*never true”* to 5 = “*very often true*”) can be summarized to the five subscales “emotional abuse”, “sexual abuse”, “physical abuse”, “emotional neglect”, and “physical neglect” and a total sum score (range from 25 to 125 by exluding 3 items on minimization/denial), with higher scores indicating more traumatization. Previous studies have shown robust psychometric properties of the CTQ with acceptable to high internal consistencies of the subscales (except for physical neglect) in clinical [74] and healthy [75] as well as community [73] samples. In our sample, a Cronbach’s alpha of 0.89 indicated good internal consistency of the total scale.

The German version of the Difficulties in Emotion Regulation Scale (DERS) [13] assesses emotion dysregulation. Its 36 items (a Likert scale from 1 = “*almost never*” to 5 = “*almost always*”) can be summarised in six subscales reflecting distinct facets of emotion dysregulation (“nonacceptance of emotional responses”, “difficulties engaging in goal-directed behaviour”, “impulse control difficulties”, “lack of emotional awareness”, “limited access to ER strategies”, and “lack of emotional clarity”) and a total score ranging from 36 to 180, with higher scores indicating higher emotion dysregulation. The DERS has demonstrated a high internal consisity for all subscales except for “lack of emotional awareness” in a clinical sample [76] and adequate reliability of all subscales in student samples [13, 77] which was confirmed by a Cronbach’s alpha coefficient of 0.97 in the current study.

The German version of the Revised Beck Depression Inventory-II (BDI-II) [78] was administered as a self-report measure to evaluate depressive symptoms. The questionnaire consists of 21 items, with participants rating their symptoms over the past two weeks on a Likert scale (0–3 points). The total score is derived by summing all item responses. The BDI-II has demonstrated high internal consistency in psychiatric and non-clinical samples [79] similarly to the current sample (α = 0.95).

### Experimental task

**Fig. 1:**
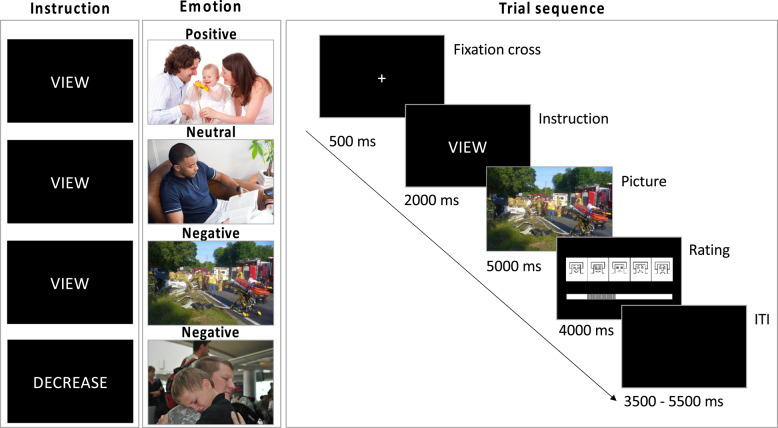
Schematic task overview. Images are resembling the IAPS pictures used. ITI, Inter-Trial Interval

The ER paradigm was an adapted version from Schönfelder et al. [20], which is based on earlier protocols by Kanske et al. [80] for use in ERP research. Similar versions of the task have been found to be effective in modulating ERPs in healthy controls [20, 80, 81], BPD patients [30] and PTSD patients [49], supporting its sensitivity to emotion regulation processes. Participants were exposed to 40 negative, 20 neutral, and 20 positive pictures in two blocks with a brief break of 2 min between blocks and a total duration of approximately 23 min. Each trial started with a fixation Cross for 500 ms followed by a one-word instruction (2000 ms) (VIEW or DECREASE). Afterwards an IAPS picture was displayed for 5000 ms after which the participants had to provide a valence rating (4000ms). A time-varying intertrial interval of 3500 to 5500 ms was displayed before the next trial sarted (see Fig. 1 for trial structure and timing).

At the beginning of the experiment, participants were instructued to either attentively look at the content of the picture (VIEW) or to actively reduce emotional reactions (DECREASE). The latter condition (DECREASE) was always followed by pictures with negative content (i.e., 20 negative pictures in the DECREASE and the VIEW condition, respectively). To achieve this, they were asked to emotionally distance themselves from the picture by trying to become an uninvolved observer and assuming that the depicted situation was only a re-enactment. Each trial ended with a rating of the current emotional valence (“*How are you feeling right now?*”) using the Self-Assessment Manikin (SAM) Scale for Valence [82], a non-verbal, pictorial 9-point scale ranging from 1= “*unpleasant*” via 5=”*neutral*” to 9=”*pleasant*” that is displayed in Fig. 1. Pictures were presented in a pseudorandomized order with no more than three pictures of the same valence and no more than six times the same instruction in a row. They were drawn from the International Affective Picture System (IAPS) [83] based on the valence ratings of the female normative IAPS sample. The 40 negative pictures displayed accidents, mutilation, loss, pain, or illness (low normative valence ratings of *M* = 1.97, *SD* = 1.30). The same set of negative pictures was presented to all participants. These images were not explicitly matched to participants’ trauma histories, and were therefore considered trauma-unrelated. However, we cannot rule out that some content (e.g., scenes of accidents) may have resembled aspects of individual trauma experiences. The 20 neutral pictures depicted individuals engaged in everyday activities (e.g., working) as well as streets with pedestrians or offices (moderate valence ratings of *M* = 5.27, *SD* = 1.40) and the 20 positive pictures showed romantic couples, scenes with happy families or children, successful athletes, and activities (high normative valence ratings of *M* = 7.71, *SD* = 1.52). All three valence categories included pictures of humans among others. In our total sample, the mean valence ratings of negative pictures were slightly higher (*M* = 3.17, *SD* = 0.94) and the ratings of positive pictures were slightly lower (*M* = 6.33 *SD* = 1.20) compared to the normative female IAPS samples. Neutral pictures were rated similarly (*M* = 5.55, *SD* = 0.73). For detailed information about the pictures used in this study, including IAPS identification numbers and normative ratings of valence, please refer to our supplementary material.

### Data acquisition

Stimulus presentation and acquisition of ratings of emotional valence were implemented with Presentation Software (Version 23.0, Neurobehavioral Systems). EEG data were recorded with 64 Ag/AgCI electrodes mounted in an EEG headcap with an equidistant 10–10 electrode system, average reference, and impedance < 10 kΩ (TMSi, Oldenzaal, The Netherlands). In addition, a vertical electrooculography (VEOG) with infra- and supraorbital electrodes was recorded.

### Data processing

The EEG data was processed in Brain Vision Analyzer 2.2 (Brain Products GmbH, Munich, Germany). First, we removed the peripheral electrodes AF8, FT7, FT8, FT9, FT10, T7, T8, PO9, and PO10 based on post hoc visual inspection due to excessive noise from muscular artefacts and subsequently recalculated the average reference. Next, data were down-sampled to 250 Hz and filtered with a 0.1 to 30-Hz (24 dB/oct) bandpass filter and a 50 Hz Notch filter. After a manual visual inspection for the exclusion of singular artefacts, an Independent Component Analysis (ICA) was used to correct artefacts, such as vertical, and horizontal eye-artefacts, or noise. The signal was then segmented into ERP segments from − 200ms to 5000ms relative to the picture onset and a baseline correction was applied using the − 200ms pre-stimulus period. Hereafter, a semiautomatic artefact rejection was used to remove epochs with artefacts based on the following criteria of Schönfelder et al. [20]: a maximum allowed voltage step of 50µV/ms, an absolute difference of ≤ 300µV within a trial (i.e., max. peak to min. peak difference) and a lowest allowed activity of 0.50µV within 100ms. Finally, trials were averaged within the VIEW condition for each emotion category (negative, neutral, positive) separately and in the DECREASE condition for negative pictures. All grand averages of stimulus-locked ERPs were calculated for patients with BPD, patients with PTSD, and healthy controls separately.

Relevant topographies of the P3 and LPP were determined with a nonparametric cluster-based permutation *t*-test (cluster-defining threshold *p* =.05; two-tailed; iterations = 5000) using the FieldTrip toolbox (Oostenveld et al., 2011) and aligned to prior research [16]. Permutation analysis allows for statistical tests over whole time series and electrodes, while still controlling for multiple comparisons [84]. The following electrodes and time windows were used for analysis: Fz, F1, F2, FCz, FC1, FC2, FC3, FC4, Cz, C1, C2, C4, C5, C6, CPz, CP1, CP2, CP4, CP5, CP6, Pz, P1, P2, P3, P4, P5, P5, POz, PO3, PO4, Oz, O1, O2; time windows: P3 (300–500ms) and LPP (600–1500ms). For the main effect of valence (negative vs. neutral vs. positive), a significant centroparietal cluster (CPC) was found over the following electrodes: Cz, C1, C2, CPz, CP1, CP2, CP3, CP4, Pz, P1, P2. For the main effect of ER (reappraisal vs. view negative), a fronto-central cluster (FCC) was found with the following electrodes: Fz, F1, F2, FCz, FC1, FC2, Cz, C1, C2, CPz, CP1, CP2. Finally, the mean amplitudes over the CPC and FCC clusters were extracted for the two time windows and used in subsequent statistical analysis.

### Statistical analysis

To analyze differences between the patient groups and healthy controls in self-report measures of childhood trauma and emotion dysregulation, we used Kruskal-Wallis rank sum tests due to the non-normal distribution of the data. Group differences and group-by-condition interactions in emotional valence ratings during the experimental task were analyzed with two repeated-measures analyses of variance (rmANOVAs). The first rmANOVA was analyzed with the between-subject factor group (BPD, PTSD, HC) and the within-subject factor emotion category on “view” trials (negative, neutral, positive) to assess *emotional reactivity*. The second rmANOVA was computed with the factors group (BPD, PTSD, HC) and ER condition (decrease vs. view) to assess ER. Please note that the latter analysis only included trials with negative pictures. Similar rmANOVAs were run to examine emotional reactivity (3 × 3 rmANOVAs) in centro-parietal P3 and LPP amplitudes as well as to assess ER (3 × 2 rmANOVAs) in fronto-central P3 and LPP amplitudes. Dunn Multiple Comparisons with Bonferroni correction for multiple testing were used as post-hoc tests. Finally, Pearson correlations and multiple regression analyses were computed to analyze whether childhood trauma is significantly associated with self-reported and neurophysiological data on emotional reactivity and regulation, while controlling for BPD and PTSD diagnosis and medication. A medication index was computed based on the Antidepressant Treatment History Form (ATHF) by Sackeim [85], which is a widely validated tool for the systematic assessment of antidepressant treatment trials and treatment resistance. In this study, it was used to quantify the current psychotropic medication use by assigning each medication a score from “1” to “4” based on the prescribed daily dosage. For participants taking multiple medications, individual scores were summed to create a medication index, reflecting overall medication load. This index was used in multiple regression analyses as continuous predictor. Of note, only individuals with BPD without an acute comorbid PTSD diagnosis (*n* = 34) were included in the multiple regression analysis to avoid confounding of the diagnoses. To account for potential confounding effects of comorbid PTSD within the BPD group, we also repeated all main analyses after excluding the 9 BPD participants with comorbid PTSD. Specifically, we re-ran the rmANOVAs on valence ratings and ERP amplitudes, as well as the Kruskal-Wallis tests. The exclusion of these cases did not significantly alter the pattern of the results. Detailed outputs of these control analyses are provided in the supplementary material (Table S1, S2, S3).

Statistical tests were run in IBM SPSS version 28. Statistical significance was set at *p* =.05, effect sizes are reported as proportions of explained variances (*η*^*2*^) and Hynh-Feldt sphericity corrections were applied in case of significant Mauchly-test (Hynh & Feld, 1976). Of note, Bonferroni correction was applied for all primary rmANOVAs and Kruskal-Wallis tests with post-hoc comparisons. Correlational and regression analyses were considered exploratory and are reported without correction. Sensitivity analyses (G*Power; α = 0.05, power = 0.80) indicated the study could detect effects of *η²* ≈ 0.018 (2 × 3 rmANOVA), *η²* ≈ 0.015 (3 × 3 rmANOVA), *η²* ≈ 0.068 (Kruskal-Wallis), and *R²* ≈ 0.084 (multiple regression), reflecting adequate power to detect small-to-medium effects across analyses.

## Results

### Participants

A total of *N* = 135 female participants took part in the study: 43 healthy women (HC; *M*_age_=26.56, *SD* = 4.70, range: 20–45 years), 43 women with BPD (*M*_age_=26.37, *SD* = 6.53, range: 18–46 years) and 49 women with PTSD (*M*_age_=26.35, *SD* = 6.92, range: 19–50 years). Descriptive statistics and group comparisons are presented in Table 1. Chi-square tests revealed significant associations between group and both education (*χ²*(4) = 17.23, *p* =.002) and occupational status (*χ²*(4) = 13.84, *p* =.008). BPD participants were overrepresented among those with low education and those out of the workforce (all |adj. res.| ≥ 2.0), while HC participants were more often employed and less often out of the workforce; no significant differences were found for the PTSD group. Group comparisons of the CTQ total score indicated that HCs experienced significantly less childhood trauma in comparison to both patients with BPD (*p* <.001) and patients with PTSD (*p* <.001). There was no significant difference between patients with BPD and PTSD on the CTQ total score (*p* =.447). However, patients with PTSD showed significantly more experiences of physical abuse than those with BPD (*p* =.026).

**Table 1:** Sample characteristics (*N* = 135)

Characteristics M (SD) or *N* (%)	HC (*n* = 43)	BPD (*n* = 43)	PTSD (*n* = 49)	HC vs. BPD vs. PTSD	BPD vs. PTSD
**Age (years)**^**a**^	26.56 (4.70)	26.37 (6.53)	26.35 (6.92)	*p* =.480	-
**Education**^**a**^				***p*** **=.002**	***p*** **>.05**
Low	2 (4.7%)	13 (30.2%)	5 (10.4%)		
Medium	23 (53.5%)	22 (51.2%)	33 (68.8%)		
High	18 (41.9%)	8 (18.6%)	10 (20.8%)		
**Occupation**				***p*** **=.008**	***p*** **>.05**
Not in workforce	1 (2.3%)	9 (20.9%)	3 (6.1%)		
In education	23 (53.5%)	24 (55.8%)	34 (69.4%)		
Employed	19 (44.2%)	10 (23.3%)	12 (24.5%)		
**Current comorbid disorders (n**,** %)**^**b**^					
PTSD	-	9 (21.4%)	49 (100%)	-	***p*** **<.001**
Depression	-	18 (42.9%)	26 (54.2%)	-	*p* =.284
Sleeping disorders	-	12 (28.6%)	12 (25.0%)	-	*p* =.702
Anxiety disorders	-	12 (28.6%)	7 (14.6%)	-	*p* =.105
OCD	-	1 (2.4%)	3 (6.1%)	-	*p* =.374
ADHD	-	7 (16.7%)	3 (6.3%)	-	*p* =.117
Eating disorder	-	6 (14.3%)	5 (10.4%)	-	*p* =.576
**Current psychotherapy**	-	27 (62.8%)	30 (62.5%)	-	*p* =.977
**Medication index**	-	1.37 (2.09)	1.0 (1.31)	-	*p* =.898
**BPD and PTSD Symptomatology**					
IPDE score	0.22 (0.57)	20.95 (4.37)	6.02 (4.13)	***p*** **<.001**	***p*** **<.001**
CAPS total	-	-	38.06 (9.52)	-	-
**CTQ**^c^					
Emotional abuse	6.98 (3.74)	16.52 (5.09)	17.61 (5.66)	***p*** **<.001**	*p* = 1.00
Physical abuse	5.21 (1.09)	7.67 (3.87)	9.76 (5.01)	***p*** **<.001**	***p*** **=.026**
Sexual abuse	5.17 (0.66)	8.76 (4.23)	11.55 (5.82)	***p*** **<.001**	*p* =.154
Emotional neglect	8.02 (4.41)	15.95 (4.88)	16.80 (4.86)	***p*** **<.001**	*p* = 1.00
Physical neglect	6.17 (1.94)	10.60 (4.23)	10.49 (3.71)	***p*** **<.001**	*p* = 1.00
Total	31.55 (9.56)	59.50 (16.05)	66.20 (17.19)	***p*** **<.001**	*p* =.447
**BDI-II Total**	3.16 (3.16)	27.95 (11.19)	23.79 (10.57)	***p*** **<.001**	*p* =.668

Note. *M *Mean, *SD* Standard Deviation, *HC* Healthy Controls, *BPD* Borderline Personality Disorder, *PTSD* Post Traumatic Stress Disorder, *IPDE* International Personality Disorder Examination, *CAPS-5* Clinician-Administered PTSD Scale for DSM-5, *CTQ* Childhood Trauma Questionnaire, *BDI-II* Beck Depression Inventory II, *OCD* Obsessive Compulsive Disorder, *ADHD* Attention Deficit Hyperactivity Disorder, Comorbidity and medication: counts do not add up due to multiple diagnoses and polypharmacy, respectively. Education and occupation were recoded into three levels. Education: low = no degree or middle school, medium = high school or vocational training, high = university degree. Occupation: Not in workforce = incapacity for work/pension/unemployed, in education = high school/apprentice/trainee/university student, employed = part- & fulltime

^a^ PTSD (*n* = 48); ^b^ BPD (*n* = 42), PTSD (*n* = 48); ^c^ HC (*n* = 42), BPD (*n* = 42), PTSD (*n* = 49). ^d^ HC (*n* = 41), BPD (*n* = 39), PTSD (*n* = 47). All p-values are Bonferroni-corrected for multiple comparisons

### Experimental measures of emotional reactivity and regulation

#### Emotional reactivity

**Fig. 2:**
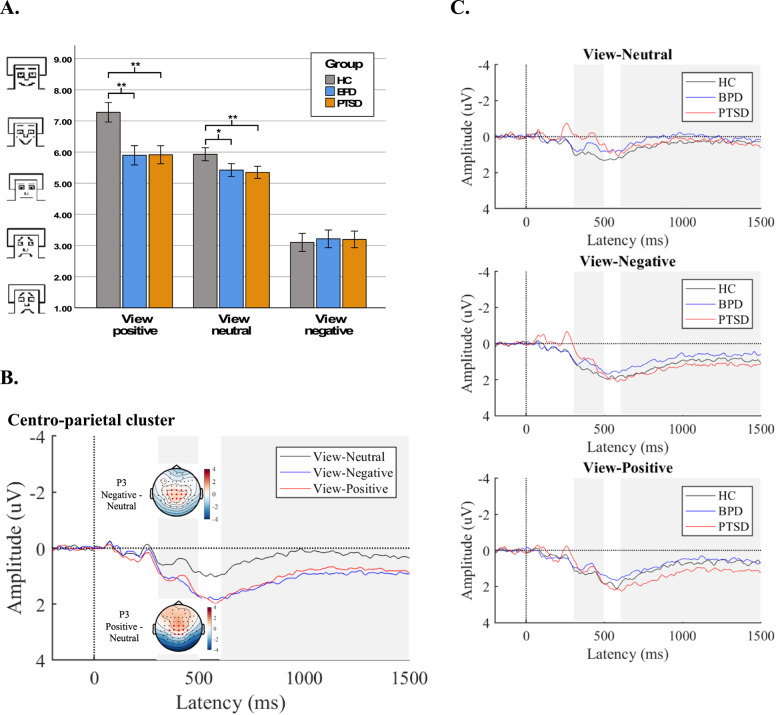
Emotional valence ratings and ERP responses for emotional reactivity. **A** Emotional valence ratings; (**B**) ERP waveforms (μV) for viewing trials with neutral, negative, and positive pictures. Topographies show the difference in activity at the P3 (300 – 500 ms) for the negative vs. neutral and positive vs. neutral trials over the cluster of centro-parietal electrodes (marked in red). **C** ERP waveforms per condition

A successful emotion induction was confirmed by a significant main effect of emotion category on *emotional valence ratings*, *F*(2,262) = 535.31, *p* <.001, *η²*_*p*_=0.80) (see Fig. 2 A). Positive pictures induced higher emotional valence (*M* = 6.33, *SD* = 1.20, *p* <.001) than neutral pictures (*M* = 5.55 *SD* = 0.73) and negative pictures (*M* = 3.17, *SD* = 0.94, *p* <.001).

A significant group effect with a large effect size (*η²*_*p*_=0.19) manifested wherein patients with BPD and PTSD exhibited decreased valence ratings compared to the control group (*F*(2,131) = 15.25, *p* <.001). Moreover, a significant group by emotion category interaction with a moderate effect size (*η²*_*p*_=0.15) was found (*F*(4,262) = 11.53, *p* <.001). According to posthoc tests (Bonferroni-corrected), patients with BPD and patients with PTSD expressed significantly lower valence ratings in response to positive (BPD < HC: *d*=−1.38, *p* <.01; PTSD < HC: *d*=−1.36, *p* <.01) as well as to neutral pictures (BPD < HC: *d*=−0.51, *p* <.05; PTSD < HC: *d*=−0.58, *p* <.01) compared to HC. There were no significant group differences in valence ratings in response to negative pictures (*p* >.05).

Visual inspection of the *neurophysiological data* (see Table 2; Fig. 2B C) indicated larger P3 and LPP amplitudes in response to negative and positive vs. neutral pictures with the most pronounced emotional effects in patients with PTSD. Confirming this, the rmANOVA revealed a significant main effect of emotion category for the centro-parietal P3 (*F*(2,264) = 39.40, *p* <.001, *η²*_*p*_=0.23) and LPP (*F*(2,264) = 42.30, *p* <.001, *η²*_*p*_=0.24) amplitudes. For P3, this effect was qualified by a significant group by emotion category interaction with a medium effect size (*F*(4,264) = 4.71, *p* =.001, *η²*_*p*_=0.07). Interestingly, group differences were particularly prominent in response to neutral pictures, where P3 amplitudes were lowest for patients with PTSD and largest for HC (PTSD < BPD: *d* = 0.54µV, *p* <.01; PTSD < HC: *d* = 0.96µV, *p* <.01; BPD < HC: *d* = 0.96µV, *p* <.01). Patients with PTSD also showed lower P3 amplitudes to negative pictures than HC (*d* = 0.51µV, *p* <.01). For the LPP, the group by emotion category interaction did not reach statistical significance (*F*(4,264) = 1.51, *p* =.201, *η²*_*p*_=0.02). No significant group effects were found in the P3 (*F*(2,132) = 1.65, *p* =.196, *η²*_*p*_=0.02) or LPP (*F*(2,132) = 2.43, *p* =.092, *η²*_*p*_=0.04).

**Table 2:** P3 and LPP amplitudes in different conditions

		HC	BPD	PTSD
		M (SD)	M (SD)	M (SD)
**P3 (CPC)**	Neutral pictures^a^	1.03 (1.25)	0.62 (1.66)	0.07 (1.58)
	Negative pictures^a^	1.48 (1.37)	1.14 (1.72)	0.97 (1.71)
	Positive pictures	1.33 (1.40)	1.01 (1.69)	1.11 (1.66)
**LPP (CPC)**	Neutral pictures	0.42 (0.93)	0.11 (1.07)	0.36 (1.19)
	Negative pictures	1.16 (1.31)	0.81 (1.25)	1.36 (1.34)
	Positive pictures	0.89 (1.27)	0.72 (1.08)	1.35 (1.34)
**P3 (FCC)**	View negative pictures	−1.63 (1.68)	−2.02 (1.99)	−2.49 (1.94)
	Regulate negative pictures	−1.33 (1.57)	−1.67 (2.15)	−2.15 (2.14)
**LPP (FCC)**	View negative pictures	0.46 (1.31)	0.30 (1.38)	0.35 (1.22)
	Regulate negative pictures	1.04 (1.39)	0.51 (1.46)	1.11 (1.57)

Note. Amplitude in µV; *M* Mean, *SD* Standard Deviation, *HC* Healthy Controls, *BPD* Borderline Personality Disorder, *PTSD* Post Traumatic Stress Disorder, *CPC* Centro-Parietal Cluster, *FCC* Frontro-Central Cluster

^a^Significant interaction effect between group and picture category (for details see text)

To summarize, both patient groups showed reduced valence ratings for positive and neutral pictures compared with healthy controls. Regarding electrophysiological measures, both patient groups exhibited significantly lower P3 amplitudes to neutral pictures, and patients with PTSD additionally showed reduced P3 amplitudes to negative pictures. No group differences were observed in LPP amplitudes.

#### Instructed emotion regulation

**Fig. 3:**
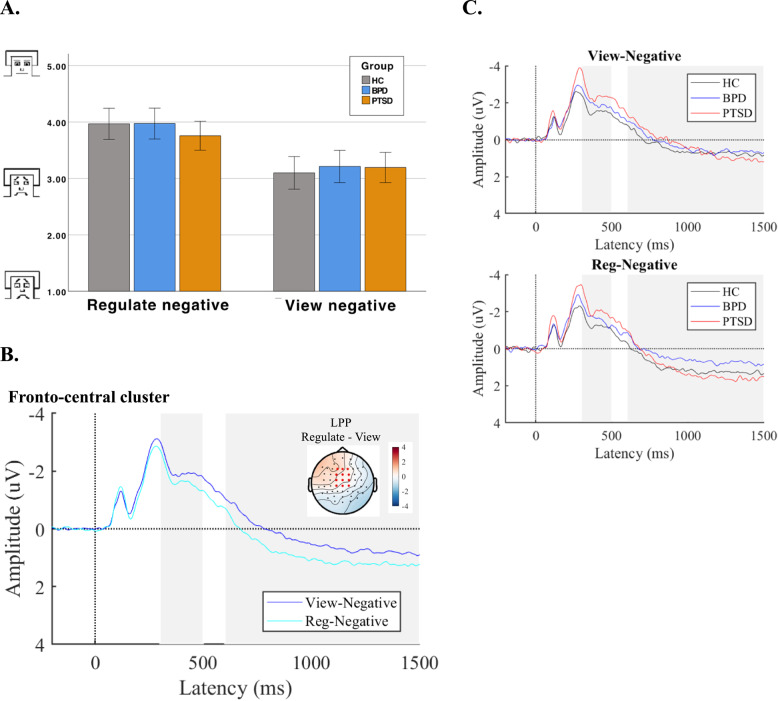
Emotional valence ratings and ERP responses for instructed ER. **A** Emotional valence ratings; (**B**) ERP waveforms (μV) on ER trials. Topographies show the difference in activity at the LPP (600 – 1500 ms) for the view negative vs. regulate negative trials over the cluster of fronto-central electrodes (marked in red). **C** ERP waveforms per condition

As displayed in Fig. 3 A, participants of all groups showed increased *ratings of emotional valence* after cognitively reappraising (*M* = 3.89, *SD* = 0.91) vs. viewing negative pictures (*M* = 3.17, *SD* = 0.94) confirmed by a significant main effect of ER (*F*(1,131) = 173.10, *p* <.001) with a large effect size (*η²*_*p*_=0.57). Not consistent with our a priori hypothesis, the group by ER condition interaction did not reach statistical significance (*F*(2,131) = 2.71, *p* =.07, *η²*_*p*_=0.04). There was no significant effect of group (*F*(2,131) = 0.21, *p* =.811, *η²*_*p*_=0.003).

Visual inspection of *neurophysiological data* (see Table 2; Fig. 3B C) suggested more positive fronto-central P3 and LPP amplitudes in the reappraisal (decrease) vs. view condition across groups. Only the main effect of ER condition reached statistical significance (P3: *F*(1,132) = 20.72, *p* <.001, *η²*_*p*_=0.14; LPP: *F*(1,132) = 26.87, *p* <.001, *η²*_*p*_=0.17), while the group ER condition interaction did not reach statistical significance (P3: *F*(2,132) = 0.04, *p* =.959, *η²*_*p*_=0.001; LPP: *F*(2,132) = 2.83, *p* =.063, *η²*_*p*_=0.04). No significant group effects were found in the P3 (*F*(2,132) = 2.31, *p* =.103, *η²*_*p*_=0.03) or LPP (*F*(2,132) = 1.04, *p* =.356, *η²*_*p*_=0.02).

Thus, across groups, instructed cognitive reappraisal of negative pictures resulted in higher emotional valence ratings and increased fronto-central ERP amplitudes (P3 and LPP). However, no significant differences were observed between the patient groups and HC in the effect of reappraisal on ratings and ERPs.

### Self-reported measures of emotion regulation

Patients with BPD and PTSD reported significantly higher emotion dysregulation than HC (see Table 3 for significant group differences for total score and all subscales, all *ps* < 0.001). Effect sizes were large for all comparisons, with *η²* values ranging from 0.34 to 0.59. Bonferroni-corrected pairwise comparisons between patients with BPD and PTSD indicated that individuals with BPD reported significantly higher total DERS scores (*p* =.013, *r* ≈.26) and greater difficulties with impulse control (*p* <.001, *r* ≈.35). Across all post-hoc comparisons, effect sizes (*r*) ranged from 0.10 to 0.35, indicating small to moderate differences.

**Table 3:** Group comparisons of self-reported emotion dysregulation (DERS)

Characteristics M (SD)	HC (*n* = 38)	BPD (*n* = 40)	PTSD (*n* = 45)	HC vs. BPD vs. PTSD *p*, η²	PTSD vs. BPD *p* (adj), *r*
Difficulties in Emotion Regulation Scale, DERS ^a^					
Non-acceptance	10.84 (4.33)	22.15 (5.18)	19.36 (6.32)	***p*** **<.001**, *η²* ≈ 0.45	*p* =.225, *r* ≈.16
Emotional awareness	13.08 (3.09)	20.59 (3.87)	18.49 (5.67)	***p*** **<.001**, *η²* ≈ 0.34	*p* =.120, *r* ≈.19
Impulse control	8.11 (2.32)	20.21 (6.03)	13.71 (5.93)	***p*** **<.001**, *η²* ≈ 0.52	***p*** **<.001**, *r* ≈.35
Emotional clarity	8.26 (3.24)	17.33 (4.08)	14.51 (4.87)	***p*** **<.001**, *η²* ≈ 0.44	*p* =.051, *r* ≈.22
ER strategies	12.79 (5.27)	27.63 (6.99)	23.89 (6.78)	***p*** **<.001**, *η²* ≈ 0.49	*p* =.216, *r* ≈.17
Goal-directed behaviour	11.21 (4.05)	20.03 (4.43)	18.58 (5.08)	***p*** **<.001**, *η²* ≈ 0.39	*p* =.785, *r* ≈.10
Total	64.29 (16.09)	127.95 (20.52)	108.53 (24.74)	***p*** **<.001**, *η²* ≈ 0.59	***p*** **=.013**, *r* ≈.26

Note. Kruskal-Wallis test statistics are reported for group comparisons. Effect sizes are reported as η² for overall tests and as r for pairwise post-hoc comparisons. Interpretation thresholds: *η²* ≥ 0.01 (small), ≥ 0.06 (medium), ≥ 0.14 (large); *r* ≥.10 (small), ≥ 0.30 (medium), ≥ 0.50 (large). Significant p-values are highlighted in bold. All p-values are Bonferroni-corrected for multiple comparisons.

### Exploratory analyses of associations between childhood trauma, emotional reactivity, and emotion regulation

We first conducted exploratory correlation analyses to examine associations between childhood trauma (CTQ total score) and emotional valence ratings, ERP amplitudes and self-reported emotion dysregulation (DERS total score) (see Table 4 for details). Across groups, childhood trauma was positively associated with lower valence ratings in response to positive (*r*=-.35, *p* <.001) and neutral (*r*=-.22, *p* =.011) pictures in the experimental task and self-reported emotion dysregulation (*r* =.61, *p* <.001) but not with any of the ERP amplitudes (all *rs*≤−0.15, *ps* ≥ 0.078).

**Table 4:** Correlations between questionnaires, emotional Valence ratings and ERP amplitudes in all participants (*N* = 135)

		1	2	3	4	5	6
1.	CTQ total	--					
2.	DERS Total	0.607**	--				
3.	Valence ratings: negative pictures	0.033	− 0.034	--			
4.	Valence ratings: neutral pictures	− 0.220*	− 0.338**	0.218*	--		
5.	Valence ratings: positive pictures	− 0.348**	− 0.498**	− 0.192*	0.611**	--	
6.	Valence ratings: cognitive reappraisal	− 0.052	− 0.201*	0.754**	0.460**	0.057	--
7.	P3 CPC view negative	− 0.085	0.045	0.121	0.060	− 0.010	0.098
8.	P3 CPC view neutral	− 0.153	− 0.019	0.079	0.108	0.078	0.092
9.	P3 CPC view positive	− 0.015	0.104	0.045	− 0.024	− 0.052	0.018
10.	LPP FCC view negative	0.008	− 0.061	0.129	0.159	0.030	0.146
11.	LPP FCC regulate negative	− 0.046	− 0.028	0.066	0.149	0.086	0.013

Note. Pearson correlation coefficients, * *p* <.05 ** *p* <.01. *CTQ* Childhood Trauma Questionnaire, *DERS* Difficulties in Emotion Regulation Scale, *CPC* Centro-Parietal Cluster, *FCC* Frontro-Central Cluster. *P*-values are not corrected for multiple comparisons.

To further explore these patterns, we conducted multiple regression analyses to examine whether childhood trauma was associated with valence ratings and self-reported emotion dysregulation, while accounting for BPD and PTSD diagnosis and medication use. When covariates were included in the model, childhood trauma was no longer significantly associated with valence ratings for positive (*β* = 0.07, 95% CI [− 0.17, 0.27], *p* =.548) or neutral pictures (*β* = 0.05, 95% CI [− 0.16, 0.31], *p* =.674). However, childhood trauma remained significantly associated with self-reported emotion dysregulation (β = 0.24, 95% CI [0.08, 0.40], *p* =.004), alongside BPD diagnosis (β = 0.78, 95% CI [0.61, 0.94], *p* <.001), PTSD diagnosis (β = 0.52, 95% CI [0.33, 0.71], *p* <.001) and medication use (β=−0.16, 95% CI [−0.28, − 0.04], *p* =.010). BPD diagnosis uniquely explained 27.5% of the variance in DERS scores, with PTSD accounting for 9.2%, and CTQ and medication for 2.8% and 2.2%, respectively.

Finally, motivated by the divergence between self-report and neurophysiological findings, we conducted additional exploratory correlations between self-report (DERS total), valence ratings and ERP measures (see Table 4). In brief, these analyses showed that negative correlations between a higher self-reported emotion dysregulation with lower valence ratings after positive (*r*=-.50, *p* <.001) and neutral pictures (*r*=-.34, *p* <.001) and lower valence after cognitive reappraisal (*r*=-.20, *p* =.026) in the experimental task. Of note, none of the questionnaires or valence ratings were significantly correlated with neurophysiological measures (all *rs*≤−0.16, *ps* ≥ 0.066).

## Discussion

The present study is the first to assess and compare emotional reactivity and regulation between patients with BPD and PTSD and healthy volunteers using a multimethodological approach. We found decreased valence ratings to positive and neutral stimuli, alongside reduced P3 amplitudes in response to neutral stimuli in both patient groups, and in response to negative stimuli in patients with PTSD suggesting an hypo-reactivity. With regard to ER, there was a striking discrepancy between self-report and experimental data: While both patient groups reported significant emotion dysregulation, they did not differ in valence ratings nor neurophysiological data (P3, LPP) in the experimental ER task. Across participants, lower emotional valence ratings for neutral and positive pictures and greater self-reported emotion dysregulation were related to childhood trauma.

### Emotional reactivity

In the experimental paradigm, participants displayed hypo-reactivity to (positive) and neutral images, as evidenced by reduced emotional valence ratings and P3 responses with large effect sizes. The groups did not differ in valence ratings in response to negative images and patients with PTSD additionally demonstrated decreased P3 amplitudes in response to negative images. These findings contradict our a priori hypothesis and previous research [30, 86]. A potential explanation could be the use of trauma-unrelated negative pictures. It has been shown that trauma-related negative stimuli (e.g., combat-related pictures) can elicit stronger P300 amplitude and lower valence ratings in PTSD patients vs. controls [32, 35, 36] and that patients with BPD and comorbid PTSD showed an emotional hypereactivity in valence ratings towards pictures with trauma-related content (i.e., sexual abuse) compared to HC [37]. This hyperreactivity towards trauma-related stimuli could be pronounced when compared to a reduced reactivity to neutral or trauma-unrelated stimuli [29, 34, 36, 41, 42, 87, 88]. Of note, since there is a lack of studies that systematically investigate this possible pattern of hyper- and hypo-reactivity in BPD and PTSD patients using ERP and valence ratings, the cited references include studies that assessed different emotional response systems, including self-reports, ERPs, heart rate, and skin conductance. In the present study, a hypo-reactivity could be confirmed, where both patient groups showed lower valence ratings for positive and neutral pictures as well as reduced P3 responses to neutral pictures.

According to Javanbakht et al. [36], this phenomenon might be a consequence of a shift in attentional resource allocation following trauma exposure. Specifically, enhanced hypervigilance towards trauma-related stimuli might come at the expense of a hypo-reactivity towards neutral, unrelated stimuli [89]. Moreover, Adenauer et al. [90] have described this dampening of emotional reactions and reduced emotional discrimination as a “vigilance-avoidance” pattern, positing it as a defence mechanism aimed at avoiding the processing of stimuli unrelated to trauma that may be overwhelming. Neurocircuitry models have supported both an under-modulation of the prefrontal cortex (PFC) resulting in a hyperreactivity of the amygdala to trauma cues as well as an over-modulation of the PFC as dissociative shutdown of trauma-unrelated emotion processing in PTSD [91]. It is also proposed that this phenomenon primarily impacts early automatic bottom-up reactivity [36], potentially explaining why group differences were observed only in the P3 component and not in the LPP, which involves more conscious processing.

### Emotion regulation

Consistent with our second hypothesis, both patient groups self-reported greater deficits in ER, which is consistent with previous literature [24, 51]. General emotion dyregulation of patients with BPD was significantly higher than of patients with PTSD. This difference between diagnostic groups was most pronounced with regard to emotional impulse control, consistent with previous research suggesting impulsivity as a core feature of BPD [92].

However, contrary to our third hypothesis there was neither a difference in valence ratings nor in neurophysiological data between the groups with regard to the effects of instructed cognitive reappraisal in the experiment. Nevertheless, these findings are in line with previous studies showing a similar discrepancy between self-report and experimental measures of emotion (dys)regulation [30, 49, 54, 93]. This divergence may stem from the assessment of different processes and timescales of ER, as supported by the weak or absent associations between these measures in the current study. McRae [14] proposed that questionnaires measure the *long-term frequency* of using ER strategies in everyday life, while experiments evaluate the *short-term success* of employing a specific strategy as instructed in a laboratory.

This brings up the clinically and theoretically relevant question if patients with BPD and PTSD exhibit deficits in their short-term ability to regulate emotions or in their long-term habitual use of adaptive strategies. Some evidence suggests that emotion dysregulation in these patient groups is not characterized by an unsuccessful, but rather an infrequent use of adaptive ER strategies [24, 93]. Consistent with our findings, patients may retain the ability to consciously regulate emotions when instructed to do so but struggle in daily life to flexibly select and apply adaptive strategies, and may instead rigidly resort to maladaptive ones [9]. This latter aspect bears clinical significance, since effective ER entails the ability to flexibly choose regulation strategies tailored to the situational context to achieve a particular goal [94]. These findings underscore the importance of combining multimethod approaches of self-report and experimental measures to capture distinct aspects of emotion processing such as dispositional tendencies and in-the-moment regulatory performance.

### Childhood trauma and emotional reactivity and regulation

In exploratory analyses, childhood trauma was associated with lower valence ratings for positive and neutral pictures, which, however, did not remain significant after controlling for diagnoses and medication. Moreover, no significant associations emerged between childhood trauma and ERP responses. These findings are partly in contrast with previous studies in healthy adults [63] and university students [64], which linked childhood trauma to dampened ERP responses to unpleasant vs. neutral pictures and to diminished ERP differences between positive and neutral pictures in trauma-exposed adolescents [65]. This effect was interpreted as a desensitization to emotions or reduced affective discrimination following childhood trauma, which aligns partially with our observed (uncorrected) correlation between childhood trauma and lower valence ratings. However, given the exploratory nature of our analyses and the scarcity and inconsistencies of comparable research, no firm conclusions can be drawn from our study regarding the relationship between childhood trauma and valence ratings or neurophysiological ERP components in controls and patients with BPD and PTSD.

Lastly our fourth hypothesis was confirmed, as childhood trauma remained significantly associated with self-reported emotion dysregulation after controlling for diagnoses and medication use. While this result also stems from exploratory analyses, it offers preliminary support for the transdiagnostic explanatory value of childhood trauma beyond traditional diagnostic boundaries [3, 58, 95]. Future research should further examine the biopsychosocial mechanisms connecting childhood trauma and emotion dysregulation, considering both risk and resilience factors, to improve transdiagnostic interventions [96].

### Limitations and future directions

Our study included a relatively large sample of patients with BPD and PTSD as well as healthy controls, with sensitivity analyses confirming adequate power to detect small-to-medium effects across key comparisons, lending strength to our multimethod investigation of emotional reactivity and regulation. Nevertheless, some limitations that may impact the generalizability and validity of the results warrant consideration. (1) Only female participants were included in this study, reducing the generalization of findings as gender differences have been found in ER [97]. (2) Comorbid disorders such as depression, which are highly frequent in the included patient samples, were not controlled for although they can profoundly influence emotion processing [98]. The reason for this is that negative affectivity is a key feature of BPD which is very difficult to disentangle from depressive mood. (3) Some patients with BPD had a comorbid diagnosis of PTSD, which can impact the subjective and physiological emotional reactivity [54]. However, we repeated all main analyses after excluding BPD participants with comorbid PTSD and this did not significantly change our results (see supplementary material Table S1, S2, S3). (4) Our study used DSM-5-based diagnostic tools (e.g., CAPS-5, SCID) and did not assess complex PTSD (CPTSD), so no firm conclusions can be drawn about this diagnosis. However, given the high rate of severe childhood trauma in our sample, some participants may have met ICD-11 criteria for CPTSD. As emotion dysregulation is central to CPTSD (Ford & Courtois, 2021), future research should clearly distinguish between BPD, PTSD, and CPTSD to better understand shared and distinct mechanisms underlying these disorders. (5) While our findings support a dampened reactivity to trauma-unrelated stimuli, they do not clarify whether this hypo-reactivity is linked to a hyper-reactivity toward trauma-related stimuli.

Besides addressing these limitations, future research should explore emotion reactivity and regulation abilities with multimethodological designs on multiple time scales and with multiple measures [14]. Beyond retrospective questionnaires, emotional processes should be assessed in daily life using ecological momentary assessment [99, 100]. This approach enables the sampling of emotional reactivity and regulation in everyday situations, allowing to measure the flexibility in choosing adaptive vs. maladaptive strategies, effectiveness, and temporal interactions with other time-varying factors (e.g., dissociation or symptomatology). These more ecologically valid indices of emotion processing are highly relevant to the theoretical understanding of emotional dysfunction and to the improvement of treatment for BPD and PTSD.

### Clinical implications

First, the dampened emotional reactivity to positive and neutral information in BPD and PTSD patients as well as negative trauma-unrelated information in PTSD patients, underscores the potential benefit of interventions targeting emotional discrimination [66]. Attentional training could be used to enhance the recognition of safety signals, helping to counterbalance the allocation of attentional resources to threat-related socio-emotional cues [23, 58]. Secondly, if patients use adaptive ER strategies less frequently, therapists should support patients in flexibly choosing adaptive strategies based on the situational context [5]. Encouraging the generalization and practice of these skills in personalized and everyday contexts is paramount. Lastly, in the clinical assessment of patients’ biographical histories, clinicians should remain mindful of the etiological significance of childhood trauma, which may contribute to changes in emotional reactivity and regulation [101].

## Conclusions

This study suggests that patients with BPD and PTSD demonstrate weaker emotional valence and neurophysiological reactions (i.e., P3) towards (positive) and neutral information than healthy individuals in an experimental paradigm. Moreover, although both patient groups self-reported difficulties in ER, neither exhibited impaired cognitive reappraisal skills in experimental assessments. This was evidenced by similar emotional valence ratings and ERPs (i.e. P3/LPP amplitudes) compared to controls. These discrepancies underscore the importance of using multimethodological study designs and integrating more ecologically valid measures in future research, to further elucidate emotional dynamics, develop integrative models and enhance transdiagnostic therapeutic interventions.

## Supplementary Information

Supplementary Material 1

## Data Availability

The data supporting this study’s findings are available on request from the corresponding author.

## Abbreviations

BDI-II: Beck Depression Inventory II
BPD: Borderline Personality Disorder
CTQ: Childhood Trauma Questionnaire
CPC: Centro-Parietal Cluster
DERS: Difficulties in Emotion Regulation
ERP: Event-Related Potentials
FCC: Frontro-Central Cluster
HC: Healthy Controls
PTSD: Post-Traumatic Stress Disorder
LPP: Late Positive Potential
rmANOVAs: Repeated-Measures Analyses of Variance
