# Supplementary material for: Psychological and neurophysiological measures of emotion dysregulation in borderline personality disorder and posttraumatic stress disorder
Source: Borderline Personal Disord Emot Dysregul. 2025 Sep 10;12:37. doi: 10.1186/s40479-025-00313-3 (PMC12424224; doi:10.1186/s40479-025-00313-3)
Supplement: Supplementary file 1 — Supplementary Material 1 [file 40479_2025_313_MOESM1_ESM.docx]

**Psychological and Neurophysiological Measures of Emotion Dysregulation in Borderline Personality Disorder and Posttraumatic Stress Disorder**

**Supplementary Material**

# Isabelle Göhre^1,2*^, Sarah Back^1,2^, Simone Schütz-Bosbach^1^, Qiaoyue Ren^1^, Larissa Wolkenstein^1,2^, André Rupp^3^, Katja Bertsch^2,4^

^1^ Department of Psychology, Ludwig-Maximilians-Universität München, Germany

^2^ German Center for Mental Health (DZPG), partner site Munich, Germany

^3^University Hospital Heidelberg, Germany

^4^Department of Psychology, Julius-Maximilians-Universität Würzburg, Germany

* Corresponding Author: Isabelle Göhre, M.Sc., Dept of Psychology, LMU Munich, Germany, Leopoldstr. 13, 80802 Munich, Tel: 089-21805199, E-Mail: [isabelle.goehre@psy.lmu.de](mailto:isabelle.goehre@psy.lmu.de)

**Submitted to:** Borderline Personality Disorder and Emotion Dysregulation

**International Affective Picture System (IAPS) codes of stimuli used in the emotion regulation experiment**

**Negative pictures**

*Low normative valence ratings (M=1.97; SD=1.30)*

Codes: 2095, 2141, 2688, 2703, 2800, 2900, 3010, 3015, 3051, 3060, 3080, 3170, 3220, 3230, 3280, 3301, 3350, 3500, 6211, 6212, 6312, 6370, 6560, 6571, 6821, 6831, 6834, 6836, 6838, 9050, 9220, 9254, 9410, 9421, 9428, 9429, 9530, 9635.1, 9910

**Neutral pictures**

*Moderate normative valence ratings (M=5.27; SD=1.40)*

Codes: 2102, 2191, 2381, 2396, 2480, 2514, 2579, 2593, 2595, 2745.1, 2749, 2840, 2850, 5410, 5470, 7493, 7550, 7700

**Positive Bilder**

*High normative valence ratings (M=7.71; SD=1.52)*

Codes: 2030, 2058, 2071, 2208, 2209, 2216, 2310, 2340, 2352, 2360, 4623, 4626, 8120, 8350, 8420, 8490, 8496, 8540

**Control analyses**

Since 21.4% (*n* = 9) of the BPD patients also had a co-morbid diagnosis of PTSD, we wanted to check if potential confounding effects influenced the results and therefore repeated all main analyses after excluding these participants (*N* = 126).

**Experimental measures of emotional reactivity and regulation**

***Emotional reactivity***

Table S1 presents the results of the 3×3 rmANOVAs examining main and interaction effects of emotion (positive, neutral, negative) and group (HC, BPD, PTSD) on valence ratings and ERP amplitudes (P3 and LPP). The pattern of results mirrored the main analyses. For valence ratings, there were significant main effects of group and emotion (both *p*<.001), as well as a group × emotion interaction (*p*<.001): BPD and PTSD participants reported lower emotional valence after viewing positive and neutral images than HC participants, with no differences for negative images. ERP analyses revealed significant main effects of emotion for both P3 and LPP amplitudes (*p*<.001). P3 amplitudes were reduced in BPD and PTSD participants in response to neutral images, and in PTSD participants also to negative images, compared to HC.

| **Table S1** Statistics of the 3x3 rmANOVAs and post-hoc comparisons for valence ratings and P3 and LPP amplitude for the effect of emotion (positive, neutral, negative) | | | | | |
| --- | --- | --- | --- | --- | --- |
| Dependent Variable | Effect | F(df) | p-value | *η²ₚ* | Post-hoc comparisons  (Bonferroni corrected) |
| Valence Ratings | Group | *F*(2, 122) = 14.48 | **<.001** | .192 | HC > BPD: *d*=0.55, *p*<.001; HC > PTSD: *d*=0.62, *p*<.001 |
|  | Emotion | *F*(2, 244) = 476.40 | **<.001** | .796 | Pos > Neu: *d*=0.81, Pos > Neg: *d*=3.17; Neu > Neg, *d*=2.36;  all *ps*<.001 |
|  | Group × Emotion | *F*(4, 244) = 11.41 | **<.001** | .158 | Pos: BPD < HC: *d*=-1.35, *p*<.01; PTSD < HC: *d*=-1.36, *p*<.01;  Neu: BPD < HC: *d*=-0.51, *p*<.05; PTSD < HC: *d*=-0.58, *p*<.05 |
| P3 Amplitude | Group | *F*(2,123) = 1.85 | .161 | .029 |  |
|  | Emotion | *F*(2, 246) = 34.74 | **<.001** | .220 | Pos > Neu, *d*= 0.56μV, *p*<.001  Neg> Neu, *d*= 0.59μV, *p*<.001 |
|  | Group × Emotion | *F*(4,246) = 4.99 | **<.001** | .075 | Neu: PTSD < HC: *d*=-0.96μV, *p*<.01;  PTSD < BPD: *d*=-0.83μV, *p*<.01;  Neg: PTSD < HC: *d*= -0.51μV, *p*<.01 |
| LPP Amplitude | Group | *F*(2,123) = 1.60 | .206 | .025 |  |
|  | Emotion | *F*(2, 246) = 36.62 | **<.001** | .229 | Pos > Neu, *d*=0.66μV, *p*<.001;  Neg> Neu, *d*=0.78μV, *p*<.001 |
|  | Group × Emotion | *F*(4,246) = 1.80 | .129 | .028 |  |
| Note. Significant effects (*p*<.05) are reported in bold. *d*, mean difference | | | | | |

***Instructed emotional regulation***

Table S2 presents the results of the 2×3 rmANOVAs examining the effects of instructed emotion regulation (view vs. regulate) and group (HC, BPD, PTSD) on valence ratings and ERP amplitudes (P3 and LPP). As in the main analysis, significant main effects of regulation were found for valence ratings, P3, and LPP amplitudes (all *ps*<.001). No significant group effects or group × regulation interactions emerged, suggesting that BPD and PTSD patients did not differ from HC in the effect of instructed emotion regulation on valence ratings or ERPs (P3, LPP).

| **Table S2** Statistics of the 2x3 rmANOVAs and post-hoc comparisons for valence ratings and P3 and LPP amplitude for the instructed emotion regulation effect | | | | | |
| --- | --- | --- | --- | --- | --- |
| Dependent Variable | Effect | F(df) | p-value | η² (partial) | Post-hoc comparisons  (Bonferroni corrected) |
| Valence Ratings | Group | *F*(2,122) = 0.54 | .586 | .009 |  |
|  | Regulation | *F*(1,122) = 149.47 | **<.001** | .551 | Reg > View, *d*=0.72, *p*<.001 |
|  | Group × Regulation | *F*(2,122) = 2.55 | .083 | .040 |  |
| P3 Amplitude | Group | *F*(2,123) = 2.74 | .068 | .043 |  |
|  | Regulation | *F*(1,123) = 24.30 | **<.001** | .165 | Reg > View, *d*=0.38μV, *p*<.001 |
|  | Group × Regulation | *F*(2,123) = 0.49 | .613 | .008 |  |
| LPP Amplitude | Group | *F*(2,123) = 0.31 | .733 | .005 |  |
|  | Regulation | *F*(1,123) = 25.95 | **<.001** | .174 | Reg > View, *d*=0.52, *p*<.001 |
|  | Group × Regulation | *F*(2, 123) = 2.42 | .093 | .038 |  |
| Note. Significant effects (*p*<.05) are reported in bold. | | | | | |

**Self-reported** **measures of emotion regulation**

Table S3 shows that, consistent with the main analysis, BPD and PTSD patients reported significantly greater emotion dysregulation than HC across all DERS subscales and the total score (all *ps*<.001). Additionally, BPD patients reported greater difficulties with impulse control (*p*=.001) compared to PTSD patients. Of note, the previously significant difference in total DERS scores between BPD and PTSD patients (*p*=.013) of the main analysis (see manuscript) became non-significant in the control analysis but approached significance (*p*=.053).

| **Table S3** Group comparisons of self-reported emotion dysregulation (DERS) | | | | | |
| --- | --- | --- | --- | --- | --- |
| **Characteristics *M (SD)*** | **HC**  **(*n* = 38)** | **BPD**  **(*n* = 31)** | **PTSD**  **(*n* = 45)** | **HC vs. BPD vs. PTSD** | **PTSD vs. BPD** |
| **Difficulties in Emotion Regulation Scale, DERS** | | | | | |
| Non-acceptance | 10.84 (4.33) | 21.61 (5.32) | 19.36 (6.32) | ***p*< .001** | *p*=.576 |
| Emotional awareness | 13.08 (3.09) | 21.26 (3.98) | 18.49 (5.67) | ***p*<.001** | *p*=.076 |
| Impulse control | 8.11 (2.32) | 20.13 (5.83) | 13.71 (5.93) | ***p*<.001** | ***p*=.001** |
| Emotional clarity | 8.26 (3.24) | 17.58 (4.11) | 14.51 (4.87) | ***p*<.001** | *p*=.073 |
| ER strategies | 12.79 (5.27) | 27.29 (7.24) | 23.89 (6.78) | ***p*<.001** | *p*=.426 |
| Goal-directed behaviour | 11.21 (4.05) | 18.97 (4.32) | 18.58 (5.08) | ***p*<.001** | *p*=1.00 |
| Total | 64.29 (16.09) | 126.84 (21.53) | 108.53 (24.74) | ***p*<.001** | *p*=.053 |
| Note. Significant p-values are highlighted in bold. Bonferroni corrected. | | | | | |
